# Microporation-Mediated Transdermal Delivery of In Situ Gel Incorporating Etodolac-Loaded PLGA Nanoparticles for Management of Rheumatoid Arthritis

**DOI:** 10.3390/pharmaceutics16070844

**Published:** 2024-06-21

**Authors:** Heba M. El Sorogy, Sahar M. Fayez, Islam A. Khalil, Gehad A. Abdel Jaleel, Ahmed M. Fayez, Hesham A. Eliwa, Hoda E. Teba

**Affiliations:** 1 Department of Pharmaceutics, College of Pharmaceutical Sciences and Drug Manufacturing, Misr University for Science and Technology, 6th of October 12566, Giza, Egypt; heba.moner@must.edu.eg; 2 Department of Pharmaceutics and Industrial Pharmacy, Faculty of Pharmacy, October 6th University, 6th of October 12566, Giza, Egypt; saharmfayez@o6u.edu.eg; 3 Department of Pharmacology, National Research Center, Dokki 12622, Giza, Egypt; gehad_abougharam@yahoo.com; 4 Department of Pharmacology and Toxicology, School of Life and Medical Sciences, University of Hertfordshire Hosted by Global Academic Foundation, New Administrative Capital 11835, Cairo, Egypt; a.fayez@herts.ac.uk; 5 Department of Pharmacology, College of Pharmaceutical Sciences and Drug Manufacturing, Misr University for Science and Technology, 6th of October 12566, Giza, Egypt; hesham.helmy@must.edu.eg

**Keywords:** etodolac, PLGA nanoparticles, central composite face-centered design, microporation, in situ gel, rheumatoid arthritis

## Abstract

Management of rheumatoid arthritis (RA) requires long-term administration of different medications since there has been no cure until now. Etodolac (ETD) is a nonsteroidal anti-inflammatory drug commonly used for RA management. However, its long-term administration resulted in severe side effects. This study aimed to develop a transdermal in situ gel incorporating ETD-loaded polymeric nanoparticles (NPs) to target the affected joints for long-term management of RA. Several PLGA NPs incorporating 1% ETD were prepared by nanoprecipitation and optimized according to the central composite design. The optimum NPs (F1) exhibited 96.19 ± 2.31% EE, 282.3 ± 0.62 nm PS, 0.383 ± 0.04 PDI, and −6.44 ± 1.69 ZP. A hyaluronate coating was applied to F1 (H-F1) to target activated macrophages at inflammation sites. H-F1 exhibited 287.4 ± 4.2 nm PS, 0.267 ± 0.02 PDI, and −23.7 ± 3.77 ZP. Pluronic F-127 in situ gel (H-F1G) showed complete gelation at 29 °C within 5 min. ETD permeation from H-F1G was sustained over 48 h when applied to microporated skin and exhibited significant enhancement of all permeation parameters. Topical application of H-F1G (equivalent to 8 mg ETD) to Wistarrat microporated skin every 48 h resulted in antirheumatic therapeutic efficacy comparable to commercial oral tablets (10 mg/kg/day).

## 1. Introduction

Rheumatoid arthritis (RA) is a chronic autoimmune disorder characterized by joint inflammation associated with high levels of pro-inflammatory cytokines. These cytokines activate matrix metalloproteinases that are linked to cartilage and bone degeneration. RA significantly affects the patient’s life quality as it leads to chronic arthritic pain, joint swelling, stiffness, and disability [1]. There is no recognized cure for RA at present [2]. However, nonsteroidal anti-inflammatory medications (NSAIDs), corticosteroids, disease-modifying antirheumatic drugs, and biological disease-modifying antirheumatic medicines are the main approaches being utilized for treatment [3]. Both glucocorticoids and NSAIDs have been used for the management of pain, stiffness, and inflammation. Moreover, disease-modifying antirheumatic and biological disease-modifying antirheumatic medicines have been used in treatment for the purpose of inhibiting specific immune and inflammatory molecules involved in joint destruction [4]. Due to some concerns about NSAIDs and glucocorticoids, including their limited efficacy, inability to modify RA in the long term, and their widespread adverse effects such as gastrointestinal and cardiac toxic effects, bone thinning, weight gain, diabetes, and immunosuppression, they both have lost their historical role as the first-line treatment [5]. However, NSAIDs still play an important role in improving the patient’s life quality by controlling the symptoms of inflammation and delaying the progress of disease. They work by inhibiting the cyclo-oxygenase (COX) enzyme, thus preventing the synthesis of prostaglandins, prostacyclin, and thromboxane, which plays a major role in the inflammatory response.

In addition to the pharmacological treatment of RA, other management guidelines have been explored in the literature. Auxiliary and alternative therapies, including polyphenolic herbal drugs [6], DMSO as an anti-inflammatory agent that modulates the production of pro-inflammatory cytokines [7], and acupuncture techniques [8], have been studied for their effectiveness in managing RA symptoms. Rehabilitation interventions, including physical and occupational therapy, have been shown to be effective in improving patient’s life quality by enhancing independence and minimizing disability [9]. Furthermore, lifestyle modification has been recognized as an efficient measure in RA management [10].

Etodolac (ETD) is one of the selective COX-II inhibitor NSAIDs used for management of RA [11]. ETD possesses some advantages over other NSAIDs by having antiproliferative and antimetastatic effects in cancer patients [12,13], which adds a significant benefit to ETD in RA treatment because arthritic patients suffer from abnormal proliferation in synovial fluid as well as metastasis of the disease. However, conventional oral therapy showed lack of specificity to the affected joints and wide systemic distribution of the drug all over the body, causing gastrointestinal disturbances which, in some cases, may lead to peptic ulcers and severe bleeding, impairment of liver and kidney functions, and other extra-articular side effects [14]. In addition, it has a half-life of about 7 h that necessitates frequent dosing [15]. Therefore, in order to avoid complications resulting from oral administration, drug targeting to the specific affected joints via an alternative route of administration is a demand in ETD use for RA treatment to reduce side effects, deliver a sufficient concentration of drug at the site of action, reduce drug dose, as well as cost, minimize dosing frequency, and improve patient compliance [3,16].

Several previous attempts had been performed to develop ETD as a topical formulation. During these attempts, ETD was formulated as cubosomes [17], emulsomes [18], niosomes [15], and nanoemulsion [19] and was loaded in lipid carriers [20], aiming to improve ETD skin permeation.

Polymeric nanoparticle drug delivery systems (NPs) have been proven to be excellent drug carriers that selectively deliver drugs to the sites of inflammation, owing to the leaky vasculature at the inflamed joints, which causes enhanced permeation and retention of NPs at the site of inflammation [21]. Furthermore, the surface of NPs can be modified by coating or binding to specific ligands that can actively target certain cell receptors that are overexpressed in arthritic joints [21]. In addition, NPs have good biodegradability and biocompatibility, can encapsulate both hydrophilic and lipophilic drugs to be released slowly, and provide a sustained release product with less frequent administration [22]. Therefore, incorporating ETD into surface-modified polymeric NPs suitable for transdermal administration can achieve the above-mentioned goals.

The transdermal route for drug delivery has many advantages as it can sustain the action of many drugs, prevent drug inactivation either by first-pass metabolism or gastrointestinal pH and enzymes, and show dose flexibility, patient acceptability, ease of application, improved drug bioavailability, and reduced side effects [2]. However, transdermal drug delivery faces the challenge of permeation through the stratum corneum (SC), the outermost hard layer of skin and the main physical barrier to most substances [23]. Microporation is a physical method used to perturb the skin barrier by creating microchannels in localized areas in the SC, thus facilitating the transdermal delivery of various drugs to deeper skin layers [24]. The created microchannels are superficial precise piercings in the SC. Therefore, they provide minimally invasive, painless, and safe modification of the skin barrier compared to hypodermic needles [25,26]. Skin microporation could be achieved through different techniques such as mechanical microneedles, thermal ablation, radiofrequency, electroporation, ultrasound, lasers, and high-pressure jets [27].

In situ hydrogel microneedles (MNs) were first investigated by Sivaraman et al. using Pluronic F-127 as a thermosensitive polymer [28]. In situ-formed microneedles utilize the sol–gel transition property of the Pluronic F-127 polymer because its sol–gel transition temperature is near body temperature. Therefore, the application of Pluronic-based drug solutions on microporated skin resulted in the flowing of drug solution in the microchannels, which was then converted to gel by the body’s temperature. The gel in the microchannels can act as a drug reservoir that sustains the effect of the drug and overcomes the physiological barrier of the SC. In addition, it bypasses the difficulties of MN manufacturing.

**Figure 1:**
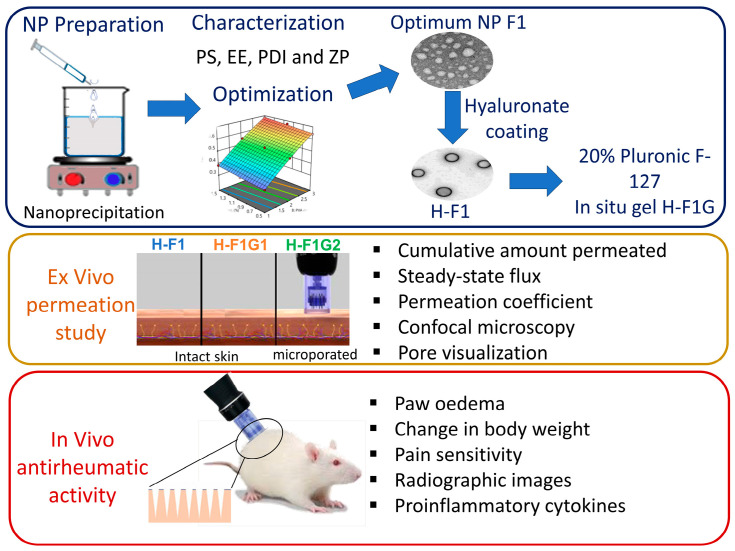
Overview of study design and different experimental phases.

The objective of this study was to develop an in situ gel incorporating ETD-loaded polymeric NPs to be administered via microporated skin to improve ETD transdermal delivery (Figure 1), target the affected joints, and perform targeted inhibition of COX enzymes within the affected joint for a prolonged period of time to reduce systemic side effects and improve patient compliance, thus improving arthritic patients’ life quality.

## 2. Materials and Methods

### 2.1. Materials

ETD was supplied as a gift from PHARCO Pharmaceuticals, Cairo, Egypt. Acid-terminated poly (lactic-co-glycolic acid) (PLGA) copolymer (Purasorb^®^ PDLG 5002A; 50/50 molar ratio of DL-lactide/glycolide copolymer) was supplied as a free sample from Purac Biomaterials, Gorinchem, The Netherlands. Polyvinyl alcohol (PVA) was purchased from BASF, Ludwigshafen, Germany. Poloxamer 407 (Pluronic^®^ acid F-127), sodium hyaluronate, complete Freund’s adjuvants (CFAs), and fluorescein sodium were obtained from Sigma Aldrich Chemical Co. (St. Louis, MO, USA). Potassium di-hydrogen phosphate, di-sodium hydrogen phosphate, and paraformaldehyde were purchased from El-Nasr Pharmaceutical Chemicals Co., Cairo, Egypt. HPLC-grade acetonitrile was purchased from SDFCL-SD Fine-Chem Limited, Mumbai, India.

### 2.2. Animals

Adult female Wistar rats weighing 250 ± 10 g were maintained under standard conditions of light (a 12 h light/dark cycle), temperature (24 °C), and hygiene with free access to standard rat chow and water and allowed to acclimatize to the conditions of the environment for 7 days. All the procedures concerning animal experiments were performed in accordance with the principles for the Care and Use of Laboratory Animals and the approved protocol of the Ethical Committee, Faculty of Pharmaceutical Sciences and Drug Manufacturing, Misr University for Science and Technology, Egypt (approval number: PH27).

### 2.3. Preparation of ETD-Loaded PLGA NPs

ETD-loaded PLGA NPs were prepared by the nanoprecipitation method, which was described first by Fessi et al. [29]. Briefly, 1% ETD and different amounts of PLGA were dissolved in acetonitrile by sonication for 30 s. The drug–polymer solution was then injected into an aqueous solution containing variable amounts of PVA as a stabilizer and stirred using a magnetic stirrer (heating magnetic stirrer, Thermolyne Corp., Buffalo, NY, USA) at 600 rpm until complete removal of the organic solvent was achieved. The final volume of dispersion was adjusted to that required using distilled water.

### 2.4. Experimental Design

For optimization of ETD-loaded NPs, a 3^2^ central composite face-centered experimental design was applied using Design-Expert^®^ Software Version 13 (Stat-Ease Inc., Minneapolis, MN, USA). Briefly, PLGA concentration (A) and aqueous phase stabilizer (PVA) concentration (B) were set as the independent variables, and their impact on the properties of ETD-loaded NPs was assessed at three different levels coded as (−1, 0, +1), whereas all other formulation variables were kept constant. The design was applied to obtain mathematical relationships between the previously mentioned independent variables and three dependent variables, Y1: entrapment efficiency (EE), Y2: particle size (PS), and Y3: polydispersity index (PDI), as shown in Table 1. The design comprised 13 experimental runs representing 4 factorial points, 4 axial points, and 5 replicates of the center point, as shown in Table 2.

### 2.5. Characterization of ETD-Loaded Polymeric NPs

#### 2.5.1. Determination of EE

The EE of ETD in PLGA NPs was determined indirectly. Briefly, ETD-loaded NP dispersions were centrifuged at 20,000 rpm and 25 °C for 60 min (cooling ultracentrifuge 3–30 K, Sigma, Taufkirchen, Germany). Then, the amount of free ETD in the supernatant was determined spectrophotometrically (UV spectrophotometer; Shimadzu, Columbia, MD, USA) at 264 nm. The measurements were performed in triplicate. The results were represented as means ± SDs. The EE of ETD in the NPs was calculated by subtracting the amount of free ETD from the total amount used in preparation according to the following equation:

(1) EE=[(total amount ETD−free ETD)÷ total amount ETD] ×100

#### 2.5.2. Determination of PS, PDI, and Zeta Potential (ZP)

The average PS of ETD-loaded NPs, size distribution (PDI), and ZP (for the optimized formulation) were measured by a particle analyzer (Malvern Zetasizer Nano ZS, Ver. 6.20, Malvern, UK) based on dynamic light scattering at an angle of 173° and a temperature of 25 °C. The samples were appropriately diluted with distilled water. Each measurement was performed in triplicate, and the results were represented as means ± SDs.

### 2.6. Optimization of ETD-Loaded NPs

The desirability function of Design expert^®^ software was utilized to obtain an optimum formulation that satisfies the previously selected constraints of maximum EE with minimum PS and PDI (Table 1). For confirmation, the suggested optimum formula was prepared and evaluated for EE, PS, and PDI to objectively assess the validity of the selected models using 95% two-sided prediction intervals of the predicted values to check if the measured actual values lie within it or not [30].

### 2.7. Determination of Drug Loading Capacity (DL) of the Optimum Formulation F1

The DL was calculated indirectly by measuring the amount of free ETD in aqueous supernatant as described previously for the determination of EE. The DL of F1 was calculated according to the following equation:

(2) DL=(Total ETD−free ETD)/NP mass

### 2.8. Preparation of Hyaluronate-Coated ETD-Loaded NPs

The optimum ETD-loaded polymeric NP formulation (F1) was coated with anionic hyaluronate using the method that was described by Kreuter et al. [31] and Mondalek et al. [32] with some modifications. Briefly, 10 mL of NP dispersion (F1) was centrifuged at 13,000 rpm for 1 h. Then, NPs were resuspended in 10 mL of aqueous hyaluronate solution at 3 different concentrations, 0.1, 0.2, and 0.4% *w*/*v*. The dispersions were stirred at 600 rpm for 30 min. The resulting NPs were ultracentrifuged again at 13,000 rpm and 25 °C for 1 h, washed twice to remove excess hyaluronate, and redispersed in 10 mL of distilled water to assess the impact of coating on PS, PDI, ZP, and DL.

### 2.9. Transmission Electron Microscopy (TEM)

The morphology of F1 and the 0.1% hyaluronate-coated NP formulation (H-F1) were visualized using TEM (JEM-1230; JEOL, Tokyo, Japan) operating at 80 kV. Before examination, a drop of the NP dispersion was placed over a carbon-coated copper grid, stained with 2% *w*/*v* phosphotungistic acid solution, and allowed to dry for about 5 min.

### 2.10. Preparation of ETD-Loaded NP In Situ Hydrogel

Incorporation of the NP dispersion into the gel was carried out to facilitate transdermal application via imparting adequate viscosity to the formulation. Poloxamer copolymers are well-known thermosensitive polymers that undergo sol–gel transformation as a response to temperature variations [33]. This property is commonly used in ocular, intranasal, and parenteral preparations. However, the sol–gel transition property of Pluronic F-127 was exploited in our work to obtain an in situ gel drug reservoir within skin microchannels formed after skin microporation using a Dr. Pen Ultima A6, Dr. Pen Inc., San Jose, CA, USA.

Three different concentrations of Pluronic F-127 (15, 20, and 25% *w*/*v*) were used in the study. Briefly, 10 mL of H-F1 dispersion was centrifuged at 13,000 rpm and 25 °C for 1 h to obtain the polymeric NPs. These NPs were resuspended in 5 mL of deionized water and mixed with 5 mL of either 30, 40, or 50% Pluronic F-127 aqueous solutions to obtain 10 mL of NP dispersion containing 15, 20, or 25% *w*/*v* Pluronic F-127. These dispersions were stored at 4 °C for further investigations.

### 2.11. Evaluation of ETD-Loaded NP In Situ Hydrogel

#### 2.11.1. Evaluation of Gelation Time

The time needed for gelation of the different formulations was assessed at skin temperature (32 °C) [28] using a modified vial inversion method as described by Kolawole et al. [34]. A 3 mL sample of each formula was placed in a glass vial and incubated in a temperature-controlled water bath (Grant Instruments, Ltd., Cambridge, UK) adjusted to 32 °C. The vials were inverted every minute and assessed for gelation visually. The time at which the formulation did not flow was recorded as the gelation time.

#### 2.11.2. Evaluation of the Lowest Gelation Temperature

The lowest gelation temperature was determined for formulations prepared with 20 and 25% *w*/*v* Pluronic F-127. A method similar to that described by Gioffredi et al. was applied [35]. Briefly, 3 mL of each formulation was placed in a glass vial and subjected to a controlled temperature increase of 1 °C starting from 20 °C. Each rise in temperature was maintained for 10 min, followed by vial inversion and visual inspection for gelation.

#### 2.11.3. Determination of Drug Loading

DL was calculated using the ETD mass incorporated in 1 mL of 20% *w*/*v* Pluronic F-127 solution (entrapped ETD mass in 1 mL H-F1 dispersion) and the mass of 1 mL of ETD-loaded NP in situ gel in the following equation:

(3) DL =mass of ETD/mass of 20% w/v Pluronic solution

### 2.12. Ex Vivo Skin Permeation Study

#### 2.12.1. Skin Preparation

Dorsal skins of adult female Wistar rats were separated. The separated full-thickness skins were stored in aluminum foil at −20 °C to keep the mechanical and barrier properties of skin [36]. Skin samples were defrosted in phosphate-buffered saline (PBS), pH 7.4, and carefully shaved before use to avoid hair interference [37]. Skin samples were cut into pieces and divided into 2 groups. The first group was used as intact skin while the other set was subjected to microporation.

#### 2.12.2. Induction of Microporation

Microporation was performed with a Dr. Pen device fitted with 12 pens needle cartridge adjusted to a 0.5 mm length and operated at the minimum speed. Skin pieces were placed on parafilm (Parafilm M Laboratory film, Neenah, WI, USA) to simulate the soft tissues under the skin and avoid excess pressure [38], then stretched by the left hand, and microchannels were created in the skin by moving the Dr. Pen across the whole area for 30 s.

#### 2.12.3. Visualization of Pores

Skin surface after microporation was visualized for pores to measure the pore-to-pore distance using the dye-binding method. The dye, 1 mL (1% *w*/*v*) of methylene blue solution, was applied immediately after microporation and left for 2 min, followed by removal of excess dye using isopropyl alcoholic swabs [28,39]. The skin was then photographed using a digital camera at 10× magnification (Canon, Melville, NY, USA).

#### 2.12.4. Confocal Microscopy

Microchannels created by the Dr. Pen were visualized and characterized for depth using a confocal laser microscope (Leica Microsystems GmbH, Wetzlar, Germany). In addition, the distribution of fluorescein-labeled H-F1G among various skin layers was examined and visualized after 1 h application on either intact or microporated skin samples to study the impact of microporation on enhancing transdermal delivery. Fluorescein sodium (0.1% *w*/*w*) was dissolved in the already-prepared formulation at 4 °C until a clear solution was formed [40]. Dorsal skins (intact and microporated) were treated with fluorescein-labeled H-F1G and kept on the skin for 1 h at 37 °C; then, the SC was cleaned with soft tissue. Skin samples were fixed using 4% *w*/*v* paraformaldehyde solution and cryo-sectioned into 10 μm thick vertical sections using a Leica CM1950 Cryostat (Leica Biosystems, Buffalo Grove, IL, USA). Reflected light images were examined for the existence of microchannels and their depth, whereas transmission images of the skin samples were examined for fluorescein distribution among various skin layers. Excitation was carried out at 488 nm and emission at 530 nm.

#### 2.12.5. Ex Vivo ETD Skin Permeation

The permeation of ETD was tested using USP dissolution tester apparatus (Hanson RS-8 plus, Hanson, St. Louis, MO, USA) [41,42,43,44]. Skin was fitted in a cylindrical glass tube with an effective diffusional area of 0.785 cm^2^. The diffusion medium consisted of 50 mL of PBS, pH 7.4, to ensure sink condition. Two formulae were tested, namely, H-F1 and H-F1G. H-F1G was examined for ETD permeation through either intact (coded as H-F1G1) or microporated skin (coded as H-F1G2). At specified time intervals, 1 mL of sample was collected from the receiver compartment and replaced with an equal volume of PBS. Samples were analyzed for the amount of ETD spectrophotometrically at 264 nm. The experiment was performed in triplicate, and the results were represented as the mean ± SD. The cumulative amount of ETD permeated per unit area (µg·cm^−2^) was plotted versus time (h) to calculate different skin permeation parameters, namely, steady-state flux, Jss (µg·cm^−2^ h^−1^), and permeation coefficient, Kp (cm·s^−1^), [45,46] as follows:

(4) $$Jss=dQ/dt\;\cdot \;A\;(µg\cdot {\mathrm{cm}}^{-2}h^{-1})$$

(5) $$Kp=Jss/Co\;(\mathrm{cm}\cdot h^{-1})$$

where *dQ*/*dt* is the slope of the linear part of the permeation plot (µg·h^−1^), A is the skin surface area available for diffusion (cm^2^), and *Co* is the initial drug concentration (µg·cm^−3^).

### 2.13. In Vivo Antirheumatic Activity

Animal models are essential for understanding the pathogenesis of RA and testing novel therapeutic interventions. This study aimed to induce RA in rats, perform radiographic imaging, and assess the expression of pro-inflammatory cytokines, including tumor necrosis factor alpha (TNF-α), interleukin 6 (IL-6), and interleukin 1-beta (IL-1 β). All the procedures concerning animal experiments were performed in accordance with the principles and guidelines for the Care and Use of Laboratory Animals (8th edition, NIH Publication, 2011) and the approved protocol of the Ethical Committee, Faculty of Pharmaceutical Sciences and Drug Manufacturing, Misr University for Science and Technology.

#### 2.13.1. Induction of Arthritis and Experimental Design

Arthritis was induced in rats by subcutaneous injection of 0.1 mL of CFA at a concentration of 1 mg/mL into the plantar surface of the right hind paw [47]. A group of 24 rats was divided randomly into four groups (n = 6) as follows:

Group I: a normal control group that received distilled water.

Group II: a positive control arthritic group.

Group III: an arthritic group that received ETD orally by oral gavage (Etodolac^®^) at a dose of 10 mg/kg once daily [48,49].

Group IV: an arthritic group that received 0.94 g of H-F1G2, equivalent to 8 mg of ETD, which was applied every 48 h [50].

The treatments started immediately after the onset of arthritis symptoms, on the day after the induction of arthritis. Treatment was continued for 28 days.

#### 2.13.2. Measuring Paw Oedema

Oedema caused by the induced arthritis was assessed clinically by measuring paw volume in mL using a plethysmometer (Type 7140, Ugo Basile, Gemonio, Italy). Pre-injection values for paw volume were measured just prior to arthritic induction for each rat and used as a baseline. The progression of arthritis was evaluated on days 5, 9, 13, 18, 23, and 28, starting from the day of induction. The percentage average change in paw volume of each group was calculated as follows:

*Paw volume average change* % = (*V_t_* − *V*_0_)/*V*_0_ × 100 (6)

where *V_t_* is the paw volume at a specified time and *V*_0_ is the paw volume of the same animal before induction of arthritis (at day 0).

The impact of treatment was evaluated by calculating the percentage inhibition of paw volume by applying the following formula [51,52]:

*Percentage inhibition* = (*V_c_* − *V*)/*V_c_* × 100 (7)

where *V_c_* is the percentage average change in paw volume of the arthritic control group and *V* is the percentage average change in paw volume of the treated group; both were compared at the same time.

#### 2.13.3. Measuring the Change in Body Weight

The rats’ body weights were measured on days 0 and 28 and expressed as the percentage of change in body weight relative to the baseline weight (day 0), as shown in the following formula:

*Change of body weight* % = *(BW on day* 28 − *BW on day* 0)/*BW on day* 0 × 100 (8)

#### 2.13.4. Behavioral Assessment of Pain Sensitivity

Behavioral pain sensitivity assessment was performed through a thermal hyperalgesia test to estimate the severity of arthritis and evaluate the effect of treatments on the thermal nociceptive threshold [53]. The thermal hyperalgesia test utilizes the fact that arthritic rats suffer from hypersensitivity to heat [54]. Hypersensitivity to thermal stimulus was evaluated in all rats from different groups using a hotplate preheated at 52 ± 1 °C. The test was performed on days 0 (baseline), 7, 14, and 27. The rats were individually placed on the hotplate, and the latency to the nociceptive reaction was recorded. Several behaviors including hind paw withdrawal, licking, flicking, or jumping were considered as a first nociceptive reaction. The test was terminated if the first response was not observed for 30 s.

#### 2.13.5. Assessment of Radiographic Images

At the end of the experiment, rats were anesthetized with sodium phenobarbital (150 mg/kg, intraperitoneal), and radiographic images were obtained. The intact animal was placed on a radiographic box, 90 cm from the X-ray source. The X-ray source was set to 50 kV and 200 A of current, and a 0.5 mm aluminum filter was used to filter the beam. Images were assessed for the presence of joint space narrowing, soft tissue swelling, and bone erosion.

#### 2.13.6. Assessment of Pro-Inflammatory Cytokines

At the end of the experiments (after 28 days), animals were anesthetized, and blood samples were collected through cardiac puncture. Serum was obtained by centrifugation at 2000× *g* and 4 °C for 10 min. Serum samples were stored at −80 °C for analysis. Serum was used to assess the levels of pro-inflammatory cytokines, TNF-α, IL-1β, and IL-6 using ELISA kits (Invitrogen Corporation, Carlsbad, CA, USA) strictly in accordance with the manufacturers’ instructions.

## 3. Results and Discussion

### 3.1. Statistical Output of the Central Composite Face-Centered Design and Diagnostic Analysis

Table 2 shows the measured dependent variables (responses) for ETD-loaded NPs. After analyzing each dependent variable (Y1: EE, Y2: PS, and Y3: PDI) individually, linear regression was applied to assess the fitting of data to different order models (linear, two-factor interaction, quadratic, and cubic models). The model with the highest prediction R^2^ for each dependent variable was the one that was ultimately chosen. Summary statistics of the used models are presented in Table 1.

As shown in Table 1, adequate precision greater than 4 was observed in all responses. This indicates that the model can be used to navigate the design space as it measures the signal-to-noise ratio [55,56]. In addition, the predicted R^2^ is in reasonable agreement with the adjusted R^2^ as the difference between both is less than 0.2 for all responses. Predicted R^2^ is calculated to determine how well the used model can fit new data. However, adjusted R^2^ is used to determine how well the model fits the observed data [55].

#### 3.1.1. Model Analysis of EE

As shown in Table 2, a high EE of ETD ranging from 93.32 ± 1.22% to 96.49 ± 2.33% was observed in all prepared NPs, which could be attributed to ETD insolubility in water and its high n-octanol/water partition coefficient, which favors partitioning of the drug into the organic phase and consequently high EE. A linear model was the best model for the analysis of the obtained data, with a non-significant lack of fit (*p* = 0.7218), high adequate precision (10.5631), and a slight difference between the adjusted and predicted R^2^ values (0.1522). The calculated equation for the EE analysis was

(9) EE=94.64+1.14 A−0.3498 B

**Figure 2:**
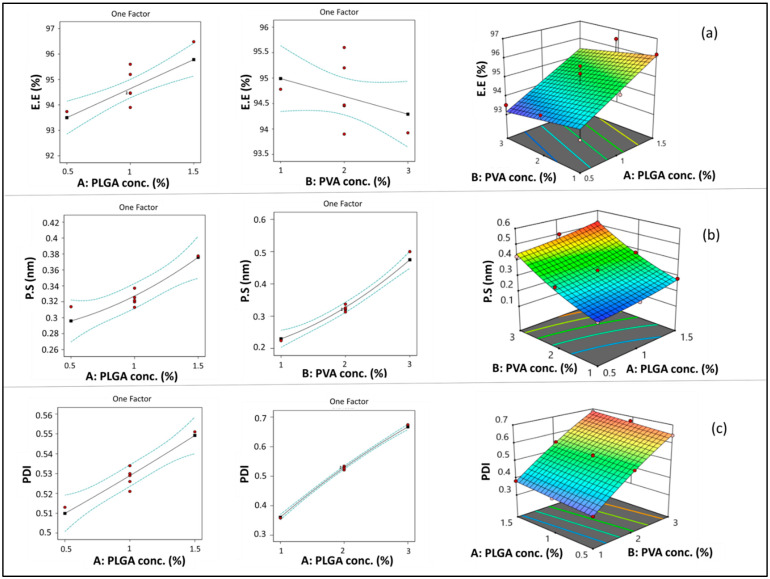
Line and response surface plots of the effects of A: PLGA concentration and B: PVA concentration on EE (**a**), PS (**b**), and PDI (**c**).

PLGA concentration (A) had a significant positive linear effect on EE (*** *p* < 0.001). Increasing PLGA concentration resulted in a significant increase in EE, as shown in Figure 2a. This could be attributed to the increased viscosity of the organic phase when increasing PLGA concentration, which consequently causes a reduction in ETD diffusion from the organic to the aqueous phase. Therefore, more drug was entrapped in the polymer matrix [57,58]. In addition, the increased PS associated with increasing PLGA concentration may result in increasing diffusional pathway lengths and decreasing drug diffusion to the aqueous phase [59].

On the other hand, although PVA concentration (B) showed a negative impact on EE (Figure 2a), the impact of this factor was non-significant (*p* > 0.05). As previously explained in previous articles when PVA concentration increased, EE decreased due to micellar solubilization [60]. However, this situation may not have a significant effect in the case of ETD due to its high lipophilicity.

#### 3.1.2. Model Analysis of PS

As shown in Table 2, the PS of different polymeric NPs ranged from 199.9 ± 1.01 to 517.5 ± 2.25 nm. Since the biological fate of NPs in the body is primarily determined by their size [61], therefore, the preparation of polymeric NPs with minimal PS is of high concern in this study. The quadratic model was the best model for analysis of the obtained data, with a non-significant lack of fit (*p* = 0.0558), high adequate precision (30.0642), and a slight difference between the adjusted and predicted R^2^ values (0.0997) (Table 1). The calculated equation for the PS analysis was

(10) $$PS=0.327+0.04\;A+0.123\;B+0.003\;AB+0.009\;A^{2}+0.025\;B^{2}$$

As shown in Figure 2b, increasing PLGA concentration (A) resulted in a significant increase in PS (*** *p* < 0.001). This result could be attributed to the increased viscosity of the organic phase by increasing polymer concentration, which opposes the effect of shearing induced by stirring and sonication. Shearing action is responsible for breaking down the PLGA solution into smaller droplets during nanoprecipitation [62]. A similar finding was observed in previous studies [60,63]. Similarly, increasing PVA concentration (B) resulted in a significant increase in the PS of the formed NPs (**** *p* < 0.0001). This result may be due to the deposition of PVA on the surface of NPs.

#### 3.1.3. Model Analysis of PDI

PDI values provide an indication of the homogeneity and size distribution of the polymeric NPs [64]. As shown in Table 2, the PDI of the prepared NPs ranged from 0.343 ± 0.02 to 0.683 ± 0.03, which revealed that ETD-loaded NPs were of adequate homogeneity. The quadratic model was the best model for the analysis of the obtained data (Table 1), with a non-significant lack of fit (*p* = 0.3023), high adequate precision (91.6582), and a slight difference between the adjusted and predicted R^2^ values (0.0061). The calculated equation for the PDI analysis was

(11) $$PDI=0.529+0.0197\;A+0.1527\;B+0.0006\;A^{2}-0.0154\;B^{2}$$

ANOVA results revealed that both PLGA concentration (A) and PVA concentration (B) had a significant positive impact on PDI (**** *p* < 0.0001) (Figure 2c). This outcome could be attributed to the increased viscosity of the organic phase by increasing PLGA concentration, which might lead to the formation of relatively larger particles with wide variation in size [65]. Furthermore, increasing PLGA concentration resulted in increased EE, which caused a consequent increase in NP heterogeneity [56,66]. Comparable results were observed with respect to PVA concentration, which may be attributed to the increased size and heterogeneity of NPs caused by deposition of PVA around its surface.

### 3.2. Optimization of ETD-Loaded NPs

For optimization, design expert^®^ software provided several solutions, representing combinations of different levels of the independent variables that best satisfied the predetermined constraints (minimum PS and PDI with maximum EE). The combination of PLGA concentration 1.5% *w*/*w* (A) and PVA concentration: 1% *w*/*w* (B) (F1) had the maximum desirability (0.844), so it was selected as the optimized formulation. The confirmation step was cancelled, as F1 (the selected optimum formula) is already one of the prepared formulations in the design with an average PS of 282.3 ± 0.62 nm, PDI of 0.383 ± 0.04, EE of 96.19 ± 2.31%, and ZP of −6.44 ± 1.69 mV.

### 3.3. Determination of DL

The DL of F1 was found to be 0.138 ± 0.0061 mg·mg^−1^.

### 3.4. Preparation of Hyaluronate-Coated NPs

To overcome the rapid clearance of PLGA NPs from blood circulation, as it limits their ability to work effectively at the sites of inflammation, hyaluronate was used as a surface-modifying agent. Hyaluronate is one of the glycosaminoglycan members that can bind CD44 receptors [67], which are overexpressed on the surface of activated macrophages that are found in arthritic joints. Hyaluronate was attached to the surface of NPs through physical association via hydrophobic interaction.

To assess the effect of hyaluronate concentration on the properties of coated NPs, some parameters, namely, PS, PDI, ZP, and DL of the obtained coated NPs, were determined, and the results are represented in Table 3. It was found that 0.2 and 0.4% hyaluronate concentrations resulted in a significant increase in PS when compared to uncoated NPs (F1) (** *p* < 0.01, *** *p* < 0.001, respectively). However, 0.1% resulted in a non-significant increase in PS (*p* > 0.05). This could be attributed to the increase in viscosity of higher concentrations of hyaluronate solutions, which consequently led to the accumulation of a thick layer on the surface of NPs that was accompanied by particle size growth. Moreover, coating NPs using 0.1% hyaluronate resulted in a significant decrease (* *p* < 0.05) in PDI, which indicates that coating with a low concentration imparts uniformity to NPs. This finding was contrary to NPs coated using higher concentrations (0.2 and 0.4%) of hyaluronate, where coated NPs showed a significant increase in PDI (*** *p* < 0.001). This result could be attributed to the higher viscosity of 0.2 and 0.4% hyaluronate solutions, which opposes uniform spreading of the coating agent over the NP surface. In addition, by comparing values of ZP of the coated and uncoated NPs, the results revealed that hyaluronate solution imparts a negative charge of magnitude directly proportional to the used concentration.

Furthermore, the change in hyaluronate concentration used for coating was found to have an impact on DL. Increasing the concentration of hyaluronate resulted in a decrease in DL. This decrease in DL may be attributed to the increased mass of the NPs due to the coating, while the amount of drug in the NPs remained constant. Additionally, the decrease in DL was found to be non-significant in the case of 0.1 and 0.2% hyaluronate solution and significant (** *p* < 0.01) in the case of 0.4% when compared to the uncoated NPs (F1).

Accordingly, ETD-loaded NPs coated using 0.1% hyaluronate were chosen for further studies and were coded as H-F1 due to their properties that enable their extravasation via leaky vasculature to the inflamed site followed by inflammatory cell sequestration [68,69], which may be facilitated by their small PS. Then, NPs could combine with CD44 on the surface of the macrophage due to hyaluronate coating to target ETD to the arthritic joint. This result could cause a reduction in systemic drug toxicity and improve the therapeutic effect of ETD by incorporating it in an inflammatory-targeted carrier.

### 3.5. Transmission Electron Microscopy

**Figure 3:**
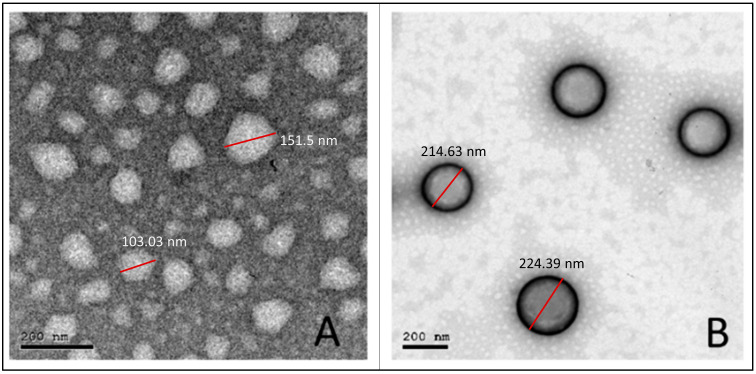
TEM micrographs for the optimized uncoated NPs (F1) (**A**) and coated NPs (H-F1) (**B**).

As shown in Figure 3, the obtained photomicrographs showed spherical particles without aggregation for both uncoated (F1) (Figure 3A) and coated (H-F1) NP formulations (Figure 3B). Furthermore, the core coat structure was recognizable for H-F1 NPs. Although particles observed from TEM micrographs showed relatively smaller sizes than those obtained from the dynamic light scattering technique, the size ratio between F1 and H-F1 NPs was in good correlation with results obtained from the Malvern zetasizer.

### 3.6. Evaluation of ETD-Loaded NP In Situ Hydrogel

#### 3.6.1. Evaluation Gelation Time

By studying the gelation time of different in situ hydrogels containing ETD-loaded NPs at 32 °C, it was found that Pluronic F-127 at a concentration of 15% did not show gelation for 10 min, while in the formulations containing 20% and 25% polymer, the time of gelation decreased when increasing the polymer concentration. The formulation containing 20% *w*/*v* Pluronic F-127 did not flow in the vial after inversion at 5 min, while the 25% polymer concentration showed complete gelation at 2 min. Accordingly, ETD-loaded NP in situ hydrogels containing 20 and 25% polymer concentrations were further investigated for the lowest gelation temperatures.

#### 3.6.2. Evaluation of the Lowest Gelation Temperature

Determination of the lowest gelation temperature is essential to select the suitable polymer concentration that remains in the sol state at RT. It allows the flow of the formulation into the microchannels created in the skin after microporation, and it is then converted into a gel inside these microchannels at 32 °C to act as a drug reservoir and bypass the SC. The lowest gelation temperature was found to be 23 °C for a polymer concentration of 25% *w*/*v* and 29 °C for 20% *w*/*v* Pluronic F-127. Therefore, 20% *w*/*v* polymer concentration was selected for further studies (coded as H-F1G) as its lowest gelation temperature is higher than RT.

#### 3.6.3. Determination of DL of the In Situ Gel Formulation (H-F1G)

The DL was found to be 0.0085 mg·mg^−1^.

### 3.7. Ex Vivo Permeation Study

#### 3.7.1. Visualization of Pores

**Figure 4:**
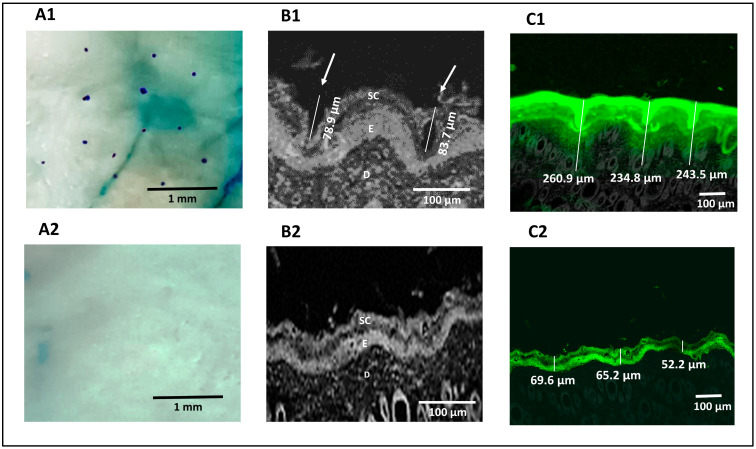
Digital images showing the pores of microchannels created in Dr. Pen-treated skin (**A1**) and absence of microchannels in untreated skin (**A2**), confocal microscopic images of Dr. Pen-treated skin demonstrating dimensions of microchannels (**B1**) and untreated skin demonstrating absence of microchannels (**B2**), confocal transmission images of fluorescein distribution in Dr. Pen-treated skin (**C1**) and untreated skin (**C2**).

The methylene blue staining technique was used for pore visualization to confirm that microporation had occurred successfully. Successful skin microporation resulted in disruption of the hydrophobic SC by creating microchannels containing hydrophilic interstitial fluid that was stained by methylene blue solution. On the other hand, intact skin around the microchannels did not take up the methylene blue stain [39,70]. Figure 4A1 showed that the application of the Dr. Pen resulted in successful microporation, confirmed by the stained microchannels. The microchannels appeared randomly scattered with an average pore-to-pore distance of 760 ± 185.3 µm (n = 10). The untreated skin (Figure 4A2) seemed unaffected by the methylene blue stain.

#### 3.7.2. Confocal Microscopy

Reflected light images obtained from a confocal microscope confirmed the formation of microchannels through the SC and viable epidermal layers in microporated skin samples, which are highlighted by white arrows in Figure 4B1. The average depth of the created microchannels was 81.3 ± 3.39 µm (n = 2). Figure 4B2 confirmed the absence of microchannels in untreated skin. Transmission confocal images of skin sections showed an intradermal distribution of fluorescein-labeled H-F1G after application for 1 h, either on intact or microporated skin. Figure 4C1 confirmed the distribution of fluorescein dye in the SC, viable epidermis, and dermis layers for an average depth of 246.4 ± 13.3 µm (n = 3) in skin samples treated with the Dr. Pen. However, the average depth of the florescent part was 62.33 ± 9.05 µm for untreated skin (Figure 4C2), which revealed that the distribution of dye was limited to the upper skin layers and failed to penetrate the deeper layers. The obtained results confirmed the impact of microchannels in enhancing transdermal permeation.

#### 3.7.3. Ex Vivo ETD Skin Permeation

**Figure 5:**
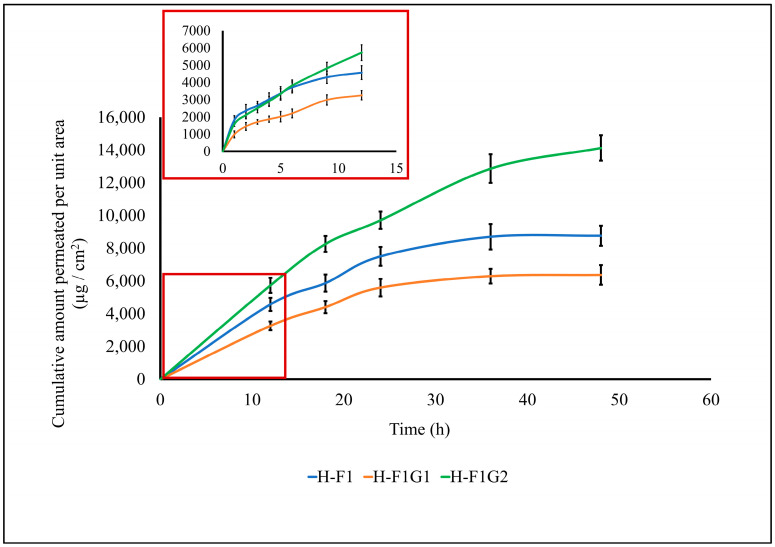
Permeation profile of ETD from different formulations. Data expressed as mean ± SD (n = 3).

Transdermal permeation of ETD from different formulations, namely, H-F1 and H-F1G1, was investigated to study the effect of formulation on permeation parameters of ETD through intact skin. Also, ETD permeation from H-F1G2 was studied to emphasize the impact of microporation on permeation enhancement. The permeation profile of ETD from the above-mentioned formulations is represented in Figure 5. The calculated permeation parameters are shown in Table 4.

By comparing the skin permeability parameters listed in Table 4, it is worth noting that the permeation of ETD was significantly enhanced (*** *p* < 0.001) from H-F1G2 compared to either H-F1 or H-F1G1 regarding the cumulative amount of drug permeated, steady-state flux (Jss), and the permeation coefficient (Kp). This confirmed the impact of microporation in improving ETD permeation. It was also found that permeation of ETD from H-F1 or H-F1G1 was diminished after 24 h, while permeation from H-F1G2 was continued for up to 48 h. In addition, about 76.84% of the loaded ETD was permeated from H-F1G2 over 48 h versus 47.68 and 34.65% from H-F1 and H-F1G1, respectively. The superior permeation of ETD from H-F1G2 could be attributed to overcoming the SC barrier, forming a drug reservoir of ETD-loaded NPs from which sustained drug permeation over a period of 48 h could be obtained. Furthermore, the beneficial properties of Pluronic F-127 caused the formulation to be applied as a liquid to flow in the formed microchannels and then converted into a gel by the effect of skin temperature from which the drug permeated over an extended period of time. This enhanced permeation creates an opportunity to develop a sustained and targeted ETD topical treatment for the management of RA.

### 3.8. In Vivo Antirheumatic Activity

#### 3.8.1. Measuring Paw Oedema

**Figure 6:**
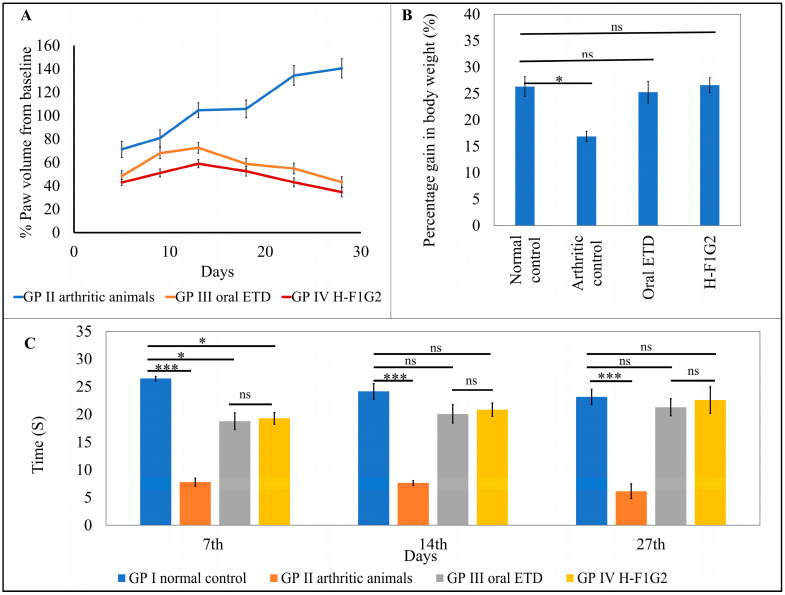
In vivo antirheumatic activity. Average percent change in paw volume of each group compared to baseline (**A**). Average gain in body weight in the experimental groups on day 28 (**B**). Time of first nociceptive response of different groups determined by thermal hyperalgesia test (**C**). Data represented as mean ± SEM, n = 6, * *p* < 0.05, *** *p* < 0.001, ns: non-significant.

The average percentage change in paw volume of each group was represented in Table 5 and Figure 6A, which illustrates the progression of RA in each group.

Figure 6A showed that the change in paw volume (% oedema) in the animals from GP III and IV was significantly smaller (* *p* < 0.05) than that which occurred in GP II, which consequently indicates that the use of ETD oral tablets or H-F1G2 slows the progression of arthritis. By applying one-way ANOVA followed by Tukey’s multiple comparisons test, the average percent change in paw volume among GP III and IV was found to be non-significant (*p* > 0.05).

The percentage inhibition of paw volume was calculated for the groups that received ETD (GP III and IV) using the arthritic non-treated rats (GP II) as a baseline to evaluate the treatment’s effectiveness in reducing the disease’s progression. According to the results in Table 5, a significant reduction (** *p* < 0.01) in rat paw volume was observed on day 28 in both GP III and GP IV by 69.3% and 75.4%, respectively.

#### 3.8.2. Measuring the Change in Body Weight

Laboratory animal body weight is utilized as a measure of pain and discomfort in addition to being a symptom of animal distress [71,72]. Body weight was nearly similar among all groups of animals at day 0 (before induction of arthritis). At the end of the experiment, on day 28, the average gain in body weight of each group was calculated. The results revealed non-significant differences in weight gain among GP I, III, and IV. On the other hand, the arthritic control group, GP II, showed a significant reduction (* *p* < 0.05) in weight gain compared to other groups by applying one-way ANOVA, as shown in Figure 6B.

#### 3.8.3. Behavioral Assessment of Pain Sensitivity

Variation in the response of rats from all experimental groups to heat appeared to be non-significant (*p* > 0.05) regarding the time of first nociceptive reaction before induction of arthritis. However, arthritic rats in GP II showed significantly lower latency time (*** *p* < 0.001) compared to normal rats in GP I by 70.6, 68.36, and 73.43% on days 7, 14, and 27, respectively. In addition, it was found that differences in latency time among GP III and GP IV were statistically non-significant (*p* > 0.05) at all test times. Moreover, by comparing GP III and GP IV to GP I (the normal control), the time of first nociceptive reaction was significantly lower than in GP I (* *p* < 0.05) by 29.11 and 27.22%, respectively, on the 7th day after induction of arthritis. On the other hand, the differences between the three groups were non- significant on the 14th and 27th days after induction of arthritis (Figure 6C). This result proved the impact of both treatments, ETD oral tablet and H-F1G2, in improving arthritic symptoms.

#### 3.8.4. Assessment of Radiographic Images

**Figure 7:**
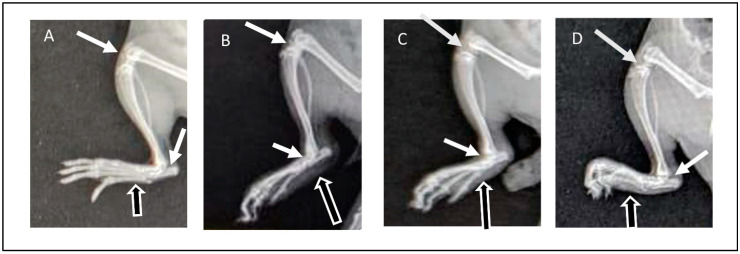
X-ray images obtained at day 28 of experiment: (**A**) normal rat (GP I), (**B**) arthritic rat (GP II), (**C**) arthritic rat that received oral ETD (GP III), and (**D**) arthritic rat treated with H-F1G2 (GP IV).

Radiographic images of the right limb were assessed for the presence or absence of soft tissue swelling (represented in figures by black arrows with white outlines) and joint space narrowing (white arrows) as well as bone thinning and erosion. The radiographic images of normal animals from GP I showed the absence of soft tissue swelling, normal joint spaces, and normal bone density without deformities or erosion (Figure 7A). Arthritic animals (GP II) showed severe inflammation demonstrated in soft tissue swelling, narrowing of joint spaces due to cartilage destruction, and thinning and erosion of paw bones (Figure 7B). On the other hand, rats from GP III that received ETD oral tablets showed a reduction in inflammatory response, demonstrated by reduced soft tissue swelling compared to rats from GP II in addition to normal bones and normal joint spaces (Figure 7C). The radiographic images of GP IV (treated with H-F1G2) showed a marked reduction in soft tissue swelling, preserved normal joint spaces, and normal bones (Figure 7D). The findings of the radiographic assessment were consistent with the results of the previously mentioned anti-inflammatory assessment tests, which confirms the superior therapeutic efficacy of H-F1G2 in the management of adjuvant-induced arthritis, owing to the novel inflammatory-targeted carrier that can provide adequate ETD concentration at the sites of inflammation over 48 h due to the formation of drug reservoirs inside microchannels. Therefore, it inhibits the release of inflammatory cytokines responsible for bone erosion and cartilage destruction.

#### 3.8.5. Assessment of Pro-Inflammatory Cytokines

It is well known that RA is associated with increased levels of pro-inflammatory cytokines secreted by activated macrophages, which are attributed to inflammation and joint tissue destruction [73]. Among several pro-inflammatory cytokines in RA, TNF-α, IL-1β, and IL-6 were selected as inflammatory markers to indicate the therapeutic efficacy of anti-arthritic treatments through assessment of their levels in serum.

**Figure 8:**
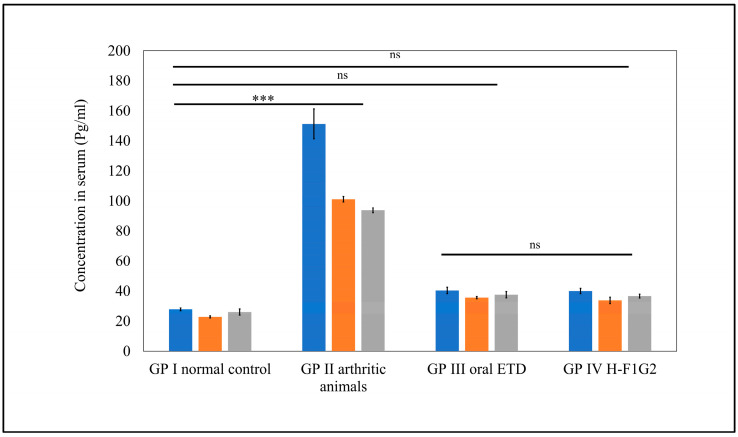
Serum levels of pro-inflammatory cytokines in different groups. Data represented as mean ± SEM, n = 5, *** *p* < 0.001, ns: non-significant.

As shown in Figure 8, significantly higher serum levels of all pro-inflammatory cytokines, namely, TNF-α, IL-1β, and IL-6, were observed in GP II (arthritic rats) (*** *p* < 0.001) compared to normal rats from GP I by 5.4-, 3.6-, and 4.4-fold, respectively. Furthermore, serum levels of pro-inflammatory cytokines in GP III and GP IV were found to be remarkably close to values of normal rats from GP I, where differences among GP I, III, and IV were found to be non-significant (*p* > 0.05) by applying one-way ANOVA followed by Tukey’s multiple comparisons test.

Interestingly, a similar therapeutic efficacy of 10 mg/kg/day ETD in the form of an oral tablet was obtained by 8 mg of ETD incorporated in H-F1G2 every 48 h. This improved therapeutic effect could be attributed to the targeting characteristics of the prepared NPs to CD44 receptors on the activated macrophages.

## 4. Conclusions

Etodolac-loaded nanoparticles were successfully prepared and optimized using a 3^2^ central composite face-centered design. The optimized formulation (F1) was subjected to hyaluronate coating (H-F1) to add targeting characteristics to CD44 receptors on activated macrophages found abundantly at inflamed arthritic joints. The thermosensitive properties of the Pluronic F-127 polymer have been exploited to prepare an in situ gel carrier incorporating H-F1 that is applied in the sol state (at RT) to microporated skin using a Dr. Pendevice and consequently flows inside the microchannels to be converted to a gel state. An etodolac-loaded nanoparticle in situ gel (H-F1G2), equivalent to 8 mg of drug administered every 2 days, exhibited superior therapeutic activity similar to an Etodolac^®^ oral tablet (at dose 10 mg/kg/day). H-F1G2 will offer a promising novel dosage form for long-term management of RA by delivering ETD to the blood circulation directly without passing GIT. Moreover, hyaluronate surface modification resulted in drug targeting towards inflamed arthritic joints, thereby reducing or eliminating severe side effects resulting from frequent peroral administration. In addition, it offers sustained drug delivery over a period of 48 h owing to the minimally invasive microporation technique that bypasses the SC and creates microchannels inside the skin that serve as reservoirs for H-F1G2 to improve patient compliance.

## Figures and Tables

**Table 1:** Independent variables with their respective levels in the 3^2^ central composite face-centered design for optimization of polymeric NPs, the desirability constraints of measured responses, model summary statistics of the used models in analysis, and the selected optimized levels for the independent variables.

Independent Variables		Level						Optimized Levels
		Low (−1)		Medium (0)		High (+1)		
PLGA (%*w*/*w*) (A)		0.5		1		1.5		1.5
PVA (%*w*/*w*) (B)		1		2		3		1
Dependent Variables	Desirability Constraints	Model	Model Summary Statistics					
			R^2^	Adjusted R^2^	Predicted R^2^	Adequate Precision	*p*-Value	Significant Factors
EE (%) (Y1)	Maximize	Linear	0.712	0.6547	0.5025	10.5631	0.002	A
PS (nm) (Y2)	Minimize	Quadratic	0.983	0.9709	0.8712	30.0642	<0.0001	A, B, B^2^
PDI (Y3)	Minimize	Quadratic	0.998	0.9974	0.9913	91.6582	<0.0001	A, B, B^2^

**Table 2:** The detailed composition of the applied 3^2^ central composite design for optimization of ETD-loaded NPs and the measured responses.

Formula No.	Space Type	PLGA Conc. (A) (%*w*/*w*)	PVA Conc. (B) (%*w*/*w*)	Y1 EE (%)	Y2 PS (nm)	Y3 PDI
F1	Factorial	1.5	1	96.19 ± 2.31	282.3 ± 0.62	0.383 ± 0.04
F2	Factorial	0.5	3	93.52 ± 1.52	424.1 ± 4.54	0.643 ± 0.03
F3	Factorial	0.5	1	93.32 ± 1.22	199.9 ± 1.01	0.343 ± 0.02
F4	Center	1	2	94.46 ± 2.14	321.6 ± 2.84	0.529 ± 0.05
F5	Center	1	2	95.2 ± 1.85	330.4 ± 2.32	0.526 ± 0.02
F6	Axial	1	3	93.93 ± 0.91	499.9 ± 5.66	0.674 ± 0.03
F7	Center	1	2	93.9 ± 1.79	337.1 ± 2.11	0.53 ± 0.03
F8	Axial	1.5	2	96.49 ± 2.33	377.5 ± 1.56	0.551 ± 0.04
F9	Center	1	2	95.6 ± 3.24	319.9 ± 3.96	0.521 ± 0.05
F10	Axial	0.5	2	93.74 ± 1.54	313.8 ± 3.12	0.513 ± 0.01
F11	Factorial	1.5	3	94.75 ± 2.47	517.5 ± 2.25	0.683 ± 0.03
F12	Center	1	2	94.47 ± 1.65	325.4 ± 5.47	0.534 ± 0.02
F13	Axial	1	1	94.78 ± 0.79	224.1 ± 1.32	0.358 ± 0.01

**Table 3:** Measured PS, PDI, ZP, and DL of coated NPs using different concentrations of hyaluronate solution.

NP	PS (nm)	PDI	ZP (mV)	DL (mg·mg^−1^)
Uncoated NPs (F1)	282.3 ± 0.62	0.383 ± 0.04	−6.44 ± 1.69	0.138 ± 0.0061
0.1% H-F1	287.4 ± 4.2	0.267 ± 0.02	−23.7 ± 3.77	0.135 ± 0.0057
0.2% H-F1	300.5 ± 5.77	0.591 ± 0.04	−27.8 ± 2.87	0.131 ± 0.0086
0.4% H-F1	439 ± 5.42	0.670 ± 0.05	−32.5 ± 5.12	0.102 ± 0.0092

**Table 4:** Comparison between different formulations in permeation parameters obtained from the permeation profile graph.

Permeation Parameter	H-F1	H-F1G1	H-F1G2
Cumulative amount of ETD permeated per unit area after 48 h (µg/cm^2^)	8764.97 ± 611.8	6368.79 ± 599.4	14,124.84 ± 777.9
Steady-state flux Jss (µg/cm^2^·h)	172.42 ± 29.75	124.79 ± 13.89	288.47 ± 7.25
Permeation coefficient Kp (cm/h)	1.79 × 10^−2^ ± 3.13 × 10^−3^	1.3 × 10^−2^ ± 1.44 × 10^−3^	3 × 10^−2^ ± 7.54 × 10^−4^

**Table 5:** Average percentage change in paw volume in GP II, GP III, and GP IV and percentage inhibition in paw volume for GP III and GP IV.

Days	Average Percentage Change in Paw Volume			Percentage Inhibition in Paw Volume	
	GP II Arthritic Animals	GP III Oral ETD	GP IV H-F1G2	GP III Oral ETD	GP IV H-F1G2
5	71.17 ± 7.02	48.22 ± 3.35	42.83 ± 2.59	32.2	39.8
9	80.86 ± 7.08	67.93 ± 4.62	50.87 ± 3.38	16	37.1
13	104.62 ± 6.46	72.53 ± 6.35	58.92 ± 3.48	30.7	43.7
18	105.73 ± 7.59	58.78 ± 4.31	52.38 ± 4.14	44.4	50.5
23	134.36 ± 8.33	54.74 ± 2.30	42.96 ± 3.85	59.3	68
28	140.41 ± 8.45	43.04 ± 3.69	34.61 ± 4.16	69.3	75.4

## Data Availability

The datasets generated and/or analyzed during the current study are available from the corresponding author on reasonable request.

## Abbreviations

RA	Rheumatoid arthritis
NSAIDS	Nonsteroidal anti-inflammatory medications
ETD	Etodolac
COX	Cyclo-oxygenase
NPs	Polymeric nanoparticle drug delivery system
SC	Stratum corneum
MN	Microneedle
CFAs	Complete Freund’s adjuvants
PVA	Polyvinyl alcohol
EE	Entrapment efficiency
PS	Particle size
PDI	Polydispersity index
ZP	Zeta potential
DL	Loading capacity
TEM	Transmission electron microscopy
PBS	Phosphate-buffered saline
TNF-α	Tumor necrosis factor alpha
IL-6	Interleukin 6
IL-1 β	Interleukin 1-beta.
